# Pathways to Care for Patients With Type 2 Diabetes and HIV/AIDS Comorbidities in Soweto, South Africa: An Ethnographic Study

**DOI:** 10.9745/GHSP-D-20-00104

**Published:** 2021-03-31

**Authors:** Edna N. Bosire, Shane A. Norris, Jane Goudge, Emily Mendenhall

**Affiliations:** a South African Medical Research Council/Wits Developmental Pathways for Health Research Unit, School of Clinical Medicine, Faculty of Health Sciences, University of the Witwatersrand, Johannesburg, South Africa.; b Global Health Research Institute, School of Human Development and Health, National Institute for Health Research, Southampton Biomedical Research Centre, University of Southampton, UK.; c Centre for Health Policy, School of Public Health, Faculty of Health Sciences, University of the Witwatersrand, Johannesburg, South Africa.; d Science, Technology, and International Affairs Program, Edmund A. Walsh School of Foreign Service, Georgetown University, Washington, DC, USA.

## Abstract

**Background:**

South Africa is experiencing colliding epidemics of HIV/AIDS and noncommunicable diseases. In response, the National Department of Health has implemented integrated chronic disease management aimed at strengthening primary health care (PHC) facilities to manage chronic illnesses. However, chronic care is still fragmented. This study explored how the health system functions to care for patients with comorbid type 2 diabetes (T2DM) and HIV/AIDS at a tertiary hospital in Soweto, South Africa.

**Methods:**

We employed ethnographic methods encompassing clinical observations and qualitative interviews with health care providers at the hospital (n=30). Data were transcribed verbatim and thematically analyzed using QSR NVivo 12 software.

**Findings:**

Health systemic challenges such as the lack of medication, untrained nurses, and a limited number of doctors at PHC clinics necessitated patient referrals to a tertiary hospital. At the hospital, patients with T2DM were managed first at the medical outpatient clinic before they were referred to a specialty clinic. Those with comorbidities attended different clinics at the hospital partly due to the structure of the tertiary hospital that offers specialized care. In addition, little to no collaboration occurred among health care providers due to poor communication, noncentralized patient information, and staff shortage. As a result, patients experienced disjointed care.

**Conclusion:**

PHC clinics in Soweto need to be strengthened by training nurses to diagnose and manage patients with T2DM and also by ensuring adequate medical supplies. We recommend that the medical outpatient clinic at a tertiary hospital should also be strengthened to offer integrated and collaborative care to patients with T2DM and other comorbidities. Addressing key systemic challenges such as staff shortages and noncentralized patient information will create a patient-centered as opposed to disease-specific approach to care.

## Abstract (precis)

Patients with type 2 diabetes are referred to tertiary hospitals in Soweto although their care could be managed at primary health care clinics. Primary health care needs to be strengthened by addressing health systemic challenges to provide integrated care for comorbid type 2 diabetes and HIV/AIDS.

## Teaser

**Key Findings:**

- Health systemic challenges such as the lack of medication, untrained nurses, and limited number of doctors at primary health care clinics necessitated patient referrals to a tertiary hospital in Soweto, South Africa.
- At the tertiary hospital, patients with multimorbidities received fragmented and uncoordinated care for their conditions.
- Little to no collaboration occurred among health care providers due to poor communication, noncentralized patient information, and staff shortage, leading to poor quality of care provided to patients.

**Key Implications:**

- Policy makers must consider primary health care clinics and providers as key actors for implementation of integrated care models. This demands more investment in the clinics in terms of equipment, medication supply, and human resources.
- Public health practitioners need to develop stronger links with communities to promote awareness on chronic diseases prevention strategies.
- More research is needed to inform the development of context-specific strategies that may enhance successful implementation of integrated care models.

## INTRODUCTION

*Nokuthula [pseudonym], seeking care at a specialty diabetes clinic in a large urban hospital, left her doctor's office with a confused look. In her mid-sixties, she had been to the clinic many times for her type 2 diabetes and other comorbid conditions, including hypertension. But this time, she had several physical complications that she needed to deal with. When she left the clinical encounter, she held a file, which she handed to the clerk at the reception, while she scrambled to manage several loose papers in her other hand. These loose papers were several referrals to other clinics, which she eventually placed on the reception desk. As the clerk was busy working on her file, Nokuthula lifted one of her loose paper, read it, and asked, “Where is the podiatrist clinic?” The clerk, without looking up, pointed to the far end, right side of clinic. Nokuthula did not understand what the clerk meant and asked again. The clerk said this time, “That room labeled Podiatry!” She then picked up the second sheet of paper and asked, “Where is the dietician clinic?” The clerk answered, “Use the stairs up to the second floor.” Then she picked up the third sheet of paper, read it, and asked; “Where is the St. Johns eye clinic?” The clerk, looking irritable, answered, “You have to walk up on the bridge from the hospital to the taxi rank just outside Bara then ask from there. The eye clinic is outside Bara. Looking confused, Nokuthula reassembled her papers and walked out.* —EB notes on the diabetes/endocrine clinic at a tertiary hospital, 2018

Living with a chronic condition such as type 2 diabetes (T2DM) and other multimorbidities—the coexistence of 2 or more chronic conditions in the same individual^1^—can pose extraordinary social and medical costs for people residing in cities such as Johannesburg, South Africa. These costs arise in part because multimorbidity is now considered a global health care priority and has become a fundamental challenge for health systems.^2^ In South Africa, a middle-income country, noncommunicable diseases (NCDs) such as T2DM have risen swiftly among communities experiencing a heavy burden of HIV as well as other infections, such as TB.^3^^–^^5^ For instance, T2DM was found to be nearly as common as HIV, TB, and hypertension in a peri-urban settlement in Cape Town, representing 45% of all consultations.^5^ In this context, patients receiving antiretroviral therapy are more likely to have T2DM than low-income people without HIV,^6^ and this risk increases exponentially the longer the patient receives antiretroviral therapy.^7^ Concurrently, the individuals most affected by this double burden of disease largely depend exclusively upon the public health system for detection, treatment, and care for both acute and chronic conditions in South Africa.^8^ Yet, public hospitals in South Africa are weak and underresourced compared with private hospitals,^9^ and they are characterized by staff shortages, inadequate equipment, and drug stock-outs,^10^ leading to longer waiting times, fewer screenings,^9^ undertreatment, and poor disease control.^11^

As a response to the disease burden, the National Department of Health in South Africa initiated the Integrated Chronic Disease Management (ICDM) model, which uses a diagonal approach to health systems strengthening.^12^ The diagonal approach integrates the vertical HIV program with the horizontal general health system.^13^ The ICDM model aims to improve the convenience and quality of chronic disease care for the majority of patients in primary health care (PHC) at a single delivery point, thereby integrating PHC into the health system.

The Integrated Chronic Disease Management (ICDM) model aims to improve the convenience and quality of chronic disease care for patients in PHC at a single delivery point, thereby integrating PHC into the health system.

Few studies have evaluated the quality of ICDM in South Africa. Despite ICDM having a greater potential to deal with the barriers experienced by patients with multiple chronic diseases, a recent study found that it has been more useful to patients with HIV because of reduced stigma, but it did not show benefits for patients with NCDs, such as hypertension.^10^ Other studies have also reported that infrastructure limitations have negatively affected the sustainability and scale-up of the model.^14^ Recently, a study conducted in Cape Town revealed that the ICDM model has not been implemented in most public PHC clinics.^15^ This has necessitated patient referral to higher levels of care that are overutilized, congested, and overburdened, leading to an escalation of health care cost.^8^^,^^16^

Care pathways are often part of integrated care and may help to guide the delivery of integrated care for patients while clarifying roles and responsibilities in the care process.^17^ In addition, due to overstretched health system, health policy makers have recommended delegation of duties, popularly known as task shifting,^18^ from physicians to other health care professionals, including nurses, pharmacists, or community health workers.^19^ As a result, tasks that have traditionally been thought of as solely within the scope of specialist practice (such as prescribing medications) can often be performed by health care workers without specialist training^18^ or occur in settings where no specialists are available. More recently, task sharing or collaborative care between different professionals, such as primary care physicians, hospital specialists, nurses, and social workers, has been considered a more appropriate strategy for managing patients with multiple chronic conditions^20^^,^^21^ and mental illnesses.^22^ This strategy is particularly important in highly skilled areas because it is difficult to shift tasks entirely to new cadres of health care workers.^23^ In this article, the terms task sharing and collaborative care are used interchangeably. We conceptualize task sharing not as a referral to other providers, such as from tertiary specialists to primary care providers, but instead a sharing of care among providers in tertiary hospitals or between tertiary hospital and primary care clinics.^19^ A good example can be drawn from a task-sharing outreach intervention program called Primary Care 101 that was implemented in South Africa to increase the capacity of nurses at primary care levels to not only take on assessment and prescribing roles for HIV and TB, but also NCDs and mental illnesses.^11^

Collaborative care has the potential to make it easier to work as a team with colleagues from other professions when managing patients, allowing better provision of patient-centered care.^24^ However, building effective partnerships requires relationships, procedures, and structures that can be different from the usual ways of working.^25^ These structures seem to be lacking in most public health care facilities in South Africa due to insufficient financial and human resources to manage the health care system.^9^^,^^11^ In addition, the failure to use electronic health records or a centralized system for patient's information in most public hospitals in South Africa^26^^–^^28^ poses a huge challenge to integrated and collaborative care for patients. But why is the ICDM model not functioning as expected?

Collaborative care has the potential to make it easier to work as a team, allowing provision of patient-centered care, but building effective partnerships requires changing the usual ways of working.

We draw from the Atun et al. conceptual framework of integration of targeted health interventions into health systems.^29^ This framework proposes that each situation uniquely affects the adoption and diffusion of new health interventions and the extent to which they are integrated into critical health system functions. Atun and colleagues identified variants as the nature of the problem being addressed, the intervention, the adoption system, the health system characteristics, and the broad context.^29^ The implementation of the ICDM model in South Africa has been slow, and it has not been scaled up to most PHC spaces. As a result, patients bypass the PHC clinics to attend hospitals for the initial contact visit, thereby increasing the cost of the service.^30^ In addition, patients with T2DM are mostly managed at secondary or tertiary levels, while HIV is managed at PHC clinics. This is partly due to limited and untrained providers at PHC clinics to properly diagnose and manage diabetes. Integrating care for chronic diseases such as T2DM into PHC clinics in South Africa can only be feasible if systemic challenges in management are addressed.^31^

We use a case study of 2 clinics that provide care for patients with T2DM, HIV/AIDS, or both at a public tertiary hospital in Soweto, South Africa, to investigate care pathways and explore how the health system functions to care for such patients. We interviewed 30 health care providers who care for patients with T2DM, HIV, or both, to better understand the challenges and opportunities within the current system for care of these conditions alone and together.

## METHODS

### Study Setting

This tertiary hospital-based study was conducted in Soweto, a peri-urban neighborhood, located about 15 km southwest of Johannesburg's central business district and with a population of approximately 1.3 million.^32^ Soweto has no designated secondary (regional or level 2) hospitals because a tertiary hospital, Chris Hani Baragwanath Academic Hospital (commonly known as Bara), provides district (level 1) and regional (level 2) hospital services, in addition to tertiary (level 3) referral services. Patients accessing care at the tertiary hospital are referred from regional, district, or PHC clinics or community clinics from outside or within Soweto. This study focuses on 2 clinics within the tertiary hospital: the medical outpatient clinic (MOPD) and the diabetes/endocrine clinic.

The MOPD covers all specialized clinics at a tertiary hospital. Apart from patients who enter the hospital through the emergency department, other patients accessing the hospital pass through the MOPD. Most patients are first managed at the MOPD and then systematically referred to specialty clinics within the hospital if specialized care is needed. The diabetes/endocrine clinic is situated opposite the MOPD. It is a specialty clinic for patients with all endocrine conditions, including diabetes.

### Study Design and Data Collection

This ethnographic study was conducted between April 2018 and December 2018 and comprised observation and semistructured interviews. All observations were conducted after obtaining consent from health care providers and patients. Observations (by EB) were conducted in different spaces of the 2 clinics: the triage room, the doctor's room, the reception area, patient queuing space, and diabetic education class. EB's role fluctuated between an observer and participant observer; she participated in everyday life of staff in the clinic by helping, watching, listening, and asking questions pertaining to care for patients. However, she did not participate in helping to manage patients clinically. She engaged in informal conversations with different people in the clinics (patients, health care providers, or caregivers). Sometimes, EB asked unstructured questions during the observations. She recorded a summary of field notes in a small jotting notebook and wrote up a full ethnographic account before the end of the day.

Thirty semistructured interviews, lasting between 30 and 60 minutes were conducted in English with health care providers to understand their experiences in managing patients with T2DM and HIV/AIDS comorbidities. The interviews included doctors, endocrinologists, dieticians, podiatrists, nurses, hospital administrators, data managers, and social workers. Initially, purposive sampling was used to select health care providers across the disciplines in the diabetes/endocrine clinic; this involved approaching existing contacts to interview and follow from within the clinic. Later, a snowball method was used to recruit more health care providers from each discipline within the diabetes/endocrine clinic (n=25). As the first author uncovered how patients navigated care in the hospital, it became apparent that some patients with T2DM were first managed at the MOPD and were only referred to the diabetes/endocrine clinic when experiencing complications. This necessitated understanding care pathways from the MOPD to the diabetes/endocrine clinic. One provider from the diabetes/endocrine clinic recommended a provider working at the MOPD, and this snow-balling method facilitated recruitment of other health care providers at the MOPD (n=5) until saturation was reached. A semistructured interview guide included questions about care for patients with T2DM and HIV/AIDS, referral system, integrated and collaborative care, barriers to care, and opportunities for care. Interviews were transcribed verbatim.

### Data Analysis

Qualitative data were thematically analyzed using Qualitative Solutions for Research (QSR) NVivo 12 software. A combined deductive and inductive approach was used for data analysis. The deductive analysis was based on the pre-identified themes focusing on the research questions and literature reviews.^4^^,^^5^^,^^10^^,^^12^^,^^15^^,^^33^ These themes included care provision for patients with chronic diseases; experience managing patients with multimorbidities; availability of resources for managing patients with multimorbidities; understanding of integrated care; collaborative care; patient-centered care; and opportunities and challenges experienced when managing patients with multimorbidities. Inductive analysis was undertaken for all themes emerging from the transcripts. EB read all transcripts repeatedly as they were developed after each interview. This process was systematic, and it facilitated re-evaluating data that were already categorized and creating new categories.^34^ These categories were refined by the constant comparative method,^35^ which involved concurrent systematic data collection and analysis. EB compared new data with previously collected data. Making comparisons facilitated challenging data that were already grouped with new categories, and this process helped in integrating the different categories and provided a holistic understanding of the phenomena under study. These categories were then reviewed by 3 other researchers involved in the study (SAN, JG, and EM) and any identified discrepancies were solved at this level. Consequently, discussions between the researchers facilitated a collaborative agreement on key emerging themes. EB then developed a codebook that was reviewed by EM. The final codebook was uploaded in QSR Nvivo 12 software where coding was done, and emerging codes were added throughout analysis. Initially, 40 parent nodes were identified, discussed, and defined. This led to reducing the parent nodes to 15, with several child nodes. Subsequent reading enabled the splitting of the parent nodes to child nodes, which provided a fast snapshot of similarities, differences, patterns, and relationships from the data. Nodes were summarized in analytical memos, and verbatim excerpts were used to report the dominant themes. The following key themes were identified: (1) organizational care pathways and the referral system; (2) managing patients with T2DM and HIV/AIDS comorbidities; and (3) patient support and involvement of family members or caregivers in care (Table 1). Case stories were developed based on observations at the clinics.

**TABLE 1.** Key Themes to Care for Patients With Type 2 Diabetes and HIV/AIDS Comorbidities, Soweto, South Africa

Theme	Expectations	Working	Not Working
Organizational—Care pathways and referral system	Multidisciplinary team working together to manage patients with comorbidities	Most patients are referred to a tertiary hospital. Most go through the medical outpatient clinic before they are referred to specialty clinics.	Limited collaboration among providers due to poor communication, staff shortage, lack of resources, and so forth.
Managing patients with type 2 diabetes and HIV/AIDS comorbidities	Efficient communication, electronic health record system	Communication is mostly done manually through a patient's file. Diabetes/endocrine clinic has implemented electronic system that captures patients' biometric data.	Due to workload and staff shortage, rarely do health providers communicate with colleagues, especially when they are in different buildings. Most other clinics use manual data capture in patient's files. Having noncentralized patient records further challenges proper communication.
Patient support and involvement of family members or caregivers in care.	Fully involve patients and their family/caregivers in care or decision making.	Mostly, patient are supported in group forums, such as during diabetes education sessions. Social workers visited patients at home.	Doctors rarely involved patients or caregivers in health care. Patient were supported in groups rather than individually. Some caregivers failed to collaborate with social workers during home visits.

In our analysis, we used the Atun et al. framework^29^ as a diagnostic tool. This process allowed a detailed mapping and understanding of how the health care system functioned in terms of care provision. Specifically, we looked at the health care intervention, which in this case was integrated care, while identifying its purpose, extent, or implementation, as well as gaps and recommendations. In other words, the framework provided a detailed mapping of chronic care at a tertiary hospital in Soweto while evaluating the purpose, extent, and nature of ICDM integration in Soweto. It also enabled us to explore what works and what does not work within the health system.

### Ethical Considerations

Written informed consent was obtained from the study participants after reading out the content of the information sheet and explaining the purpose of the study. A research committee at the tertiary hospital and the human research and ethics committee at one of the largest universities in Johannesburg approved this study (M171125).

## RESULTS

Table 2 describes the 30 health care providers who were recruited from the diabetes/endocrine unit (n=25) and the MOPD (n=5). Participants' median age was 40 years (interquartile range=15). The majority of providers had worked for more than 20 years and were trained in diverse disciplines. We discuss the 3 emergent themes in turn.

**TABLE 2.** Sociodemographic Characteristics of Study Health Care Providers in the Medical Outpatient Clinic and Diabetes/Endocrine Clinic, Soweto, South Africa

	No. (%) N=30
Gender	
Male	12 (40)
Female	18 (60)
Age, years	
25–35	9 (30)
36–45	10 (33)
46–55	8 (27)
>56	3 (10)
Profession	
Administrator	3 (10)
Data manager	3 (10)
Dietician	4 (13)
General doctor	6 (20)
Endocrinologist	3 (10)
Nurse (professional nurse, diabetes educator)	6 (20)
Podiatrist	3 (10)
Social worker	2 (6)
Years of service	
<5	5 (17)
6–10	6 (20)
11–20	9 (30)
>20	10 (33)

### Organizational Care Pathways and the Referral System

Health care providers described how most patients used 2 main structured channels in accessing the tertiary hospital: (1) a referral from PHC clinics, or (2) patient who came directly through the emergency department (Figure). One nurse described this process as follows:

*There is a referral system, patient need to start there [PHC clinics]. The doctor must write a referral letter for them to come here [tertiary hospital]. Some will come in through the emergency department. Once they are treated here, they are down referred for management and collection of pills at the community clinics.* —Provider 1, nurse

**FIGURE:**
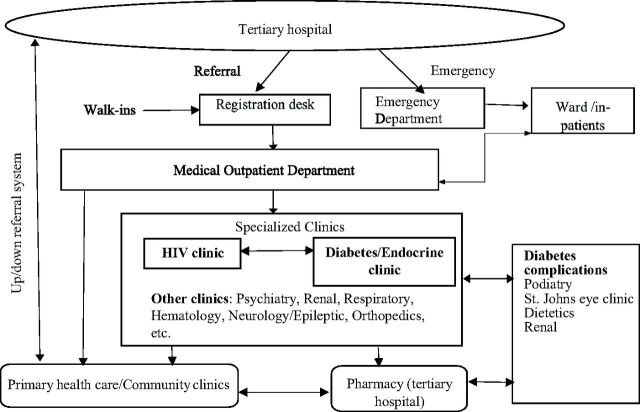
Up and Down Referral System From Primary Health Care to a Tertiary Hospital in Soweto, South Africa

#### Limitations in the Current System

Health care providers reported that the current referral system design failed to meet patient needs for various reasons. First, providers often referred patients from the PHC clinic to tertiary hospitals because of health systemic challenges (such as lacking medication, equipment, untrained nurses, and limited number of doctors) as opposed to patients needing the specialist care often associated with such referrals:

*Due to lack of necessities at primary clinics, sometimes the GP understand it's pointless to write the letter to send this patient to PHC, only for the patient to be sent back to us [a tertiary hospital].* —Provider 2, doctor

*Primary health care clinics have got limited resources; they can't do the blood tests required for diabetic care.* —Provider 3, doctor

Many argued that because the PHC clinics in Soweto were managed by nurses, as opposed to doctors who only worked in the clinics on rotational basis, many nurses were overburdened by their patient load, or untrained to manage patients with diabetes, which caused them to initiate more up-referrals from PHC to the tertiary hospital:

*Doctors come on a specific day, let's say on Wednesday. If a patient comes on Monday and it's beyond the sister's scope, they will refer the patient to tertiary hospital.* —Provider 4, nurse

*Some nurses at PHC are not well trained to diagnose patients with diabetes early enough. They only suspect that a patient is diabetic when patients are already experiencing complications, and this makes them refer them to tertiary hospital.* —Provider 11, endocrinologist

As a way of increasing nurse capacity in PHC clinics in Soweto, one doctor explained why an outreach program was important yet challenging to implement in this context:

*Our clinics at the tertiary hospital are overwhelmed by new cases of diabetes and diabetes complications because, nothing is happening at the community – where we expect prevention strategies to be taking place [… .]. I started an outreach program which I conduct alone, and on a voluntary basis. It entails educating patients at the community and, capacity building nurses who work in the PHC clinics in Soweto. I have done this exercise for a couple of years now, though it is challenging because I don't have any support in terms of finances, logistics and facilitations. Again, because there are many clinics in Soweto, I sometimes end up seeing one clinic maybe two times in a year. Therefore, we still have many patients being referred from PHC clinics to the tertiary hospital.* —Provider 2, doctor

Some opined that patients' self-referrals from PHC to the tertiary hospital were simply due to geographic convenience or perceived better quality there:

*Some patients will walk in because Bara is very close to where they live compared to a primary clinic in Soweto*. —Provider 5, hospital administrator

*They [patients] don't want to go to the local clinic, they will say there are no medication.* —Provider 6, nurse

This pattern of skipping the PHC clinic and therefore not following the traditional referral process was described as common negligence because a patient who walked in without a referral would still be allowed to access the hospital:

*The problem is that here [tertiary hospital], they cannot turn the patient away. The person at registration will give them a number and allow them in*. —Provider 2, doctor

Many providers suggested that such deference to protocol was central to reinforcing these structural factors, such as overwhelming workloads, that played a primary role in creating bottlenecks at the tertiary hospital, thus compromising patients' quality of care.

#### Down-Referral, Medication, and Challenges

The existing referral system design requires that once patients have received treatment and are stable at the tertiary hospital, they are down-referred to community or PHC clinics for management and continuous collection of their medications:

*We have a down referral form, this is what we use to send them back to the community clinics.* —Provider 1, nurse

A MOPD doctor also described how prepackaged medication is sent to local clinics:

*Medication is prepared at the chemist here [tertiary hospital] and then sent to the local clinic. Then at the local clinic, there is a chemist staff who just takes the medication out of the shelf and dispenses to the patient.* —Provider 16, doctor

Most providers reported that 6 main clinics in Soweto were selected to be central for medical supplies and refilling to patients with chronic conditions. These clinics were Lilian Ngoyi, Chiawelo, Mofolo, Stretford, Laneshia South, and Zola. Medications were distributed to these clinics and patients could select any of the 6 clinics based on which was closest to them for repeat medical supplies. This plan did not work as expected because of drug stock-outs:

*Patients will always complain that they missed their medications at the primary clinics due to drug stock-outs.* —Provider 8, nurse

Drug stock-outs at PHC clinics were attributed to various reasons. First, stock-outs were said to be a trickledown effect of drug stock-outs at the tertiary hospital, which in turn influenced nonsupply of medications to PHC clinics:

*The hospital does not pay the drug suppliers on time. If you want to confirm this, they keep on changing the suppliers because, some have stopped supplying the drugs*. —Provider 9, data manager

Oversupplying medication to patients was also linked to drug stock-outs at the tertiary hospital:

*They will issue extra medication to patient and at some point, you will be told this and that is missing from the pharmacy. This affects supply to primary clinics too* —Provider 10, doctor

In addition, the system of prepackaging medication and sending it to PHC clinics was said to be failing as described below:

*There are so many steps in the system; it could be that the courier dropped the parcel, could be someone stole the medication and so on.* —Provider 11, endocrinologist

The challenges experienced in the referral system were majorly attributed to poor communication between different providers at different levels of care:

*You imagine the patient coming here, from Orange farm to say, “I didn't get medication.” Already they are in turmoil […], poor communication is the biggest challenge in the referral process*. —Provider 12, nurse

### Managing Patients With Comorbid T2DM and HIV/AIDS

This study revealed limited integration of chronic services at the tertiary hospital. This limitation was partly because of the design of the tertiary hospital, which is specialized into different clinics. Patients with comorbid T2DM and HIV received care in 2 separate clinics: at the MOPD or diabetes/endocrine clinic for their diabetes, while those with HIV would receive care at the Nthabiseng (HIV/AIDS) clinic—a separate stand-alone clinic that was 3 minutes' walk from the building housing the diabetes/endocrine clinic. One doctor explained this:

*We don't do the HIV treatment itself here, they go to HIV clinic. Then they will come back here [diabetes/endocrine clinic] on the same day or different days for diabetes care.* —Provider 2, doctor

Another doctor said that:

*Patients go in the morning in one clinic and as soon as they are done with that clinic, they'll go and queue in the other clinic.* —Provider 10, doctor

The diabetes/endocrine clinic only managed patients with endocrine conditions:

*We only manage patients with endocrine conditions in this clinic. Patients with HIV must be managed at HIV clinic*. —Provider 2, doctor

Not unsurprisingly, individuals with both T2DM and hypertension often received care in tandem; however, for every other physical and psychiatric condition, patients would visit specific clinics. Patients with diabetes complications, such as neuropathy of the hands and feet and nephropathy, visited other, specific clinics (as illustrated in Nokuthula's case). Some of these clinics were situated outside a tertiary hospital. Although they were a very short walk from the hospital, the distance could be difficult for someone with a disability. Health care providers emphasized that collaboration and coordination of care were imperative in such instances, but they rarely occurred.

Nevertheless, providers acknowledged that collaboration or task sharing between different professionals, such as primary care providers, specialists at a tertiary hospital, nurses, and social workers, would be a good thing and would improve patients' quality of care:

*Working together means we are treating the patient well by providing what we are thinking is best for the patient. If she's amputated and she's the bread winner for example, then nothing else will be happening at home. So, with that consideration we might motivate the surgeon to do amputation that is not so intrusive.* —Provider 14, Podiatrist

Health care providers reported that the current referral system design failed to meet patient needs for various reasons.

Providers acknowledged that collaboration or task sharing between different professionals would be a good thing and would improve patients' quality of care.

They also gave examples on instances where they collaborated in caring for patients:

*Yeah, we work as a team. We [educators] will teach them about the disease, the dietician teaches about what they need to eat, then the podiatrist will teach them on the foot care.* —Provider 15, diabetes nurse educator

However, providing integrated and collaborative care for patients with HIV and T2DM was said to be difficult due to poor communication, noncentralized patient information, staff shortage, and limited resources, among other reasons (Table 3).

**TABLE 3.** Challenges to Collaborative and Integrated Chronic Care for Patients With HIV and Type 2 Diabetes in Soweto, South Africa

Theme	Excerpts
Poor communication	“There is no consistent communication between a tertiary hospital and these community clinics. Sometimes, if they [patients] see that medication is running out, they will walk to the nearest clinics, some of the clinics give them medication even without any down referral letter.” —Provider 4, nurse
	“This is something that happened last week […] at respiratory the doctor prescribed medication for her respiratory problem and diabetes as well. She went to the pharmacy and collected medication for diabetes twice in a day” —Provider 6, nurse)
Noncentralized patient information	“It's frustrating for them, isn't it? the files get lost every now and then. Patients have to queue for opening of new files, they have to figure out what medication they were on to tell the doctor…” —Provider 22, endocrinologist
	“Look, honestly until we have an electronic record keeping system in the whole hospital, record keeping is going to be in shambles and working as a team will only be a dream. Look, I only see diabetes patients twice a month which means I only use the Intellovate system twice a month, the rest of the other time I am using manual paper recording in other departments.” —Provider 23, doctor
Staff shortage, workload, and unavailability of doctors	“It is difficult because doctors have a lot on their hands […] they are expected to see a number of patients here [diabetes clinic], expected to do this and that and by the time they come back here, the queue has build up again. They will not have time for collaboration with others.” —Provider 1, nurse
Lack of resources such as medication	“The problem is not the model [ICDM] but lack of resources. […] I treated the patient and when he got well, I designed a chronic medication plan, I referred the patient to the local clinic but the patient came back and said there are no medications there.” —Provider 2, doctor
Proximity of clinics	“Collaboration is difficult. All these clinics are isolated from each other. So now, we have interprofessional communication where I write my own recommendations, you write your own recommendations, somebody writes their own without involving the patient.” —Provider 26, endocrinologist
Interprofessional conflicts	“Most of the time, surgeons will override anyone's decision. I don't know why it's like that, but sometimes they do. So […] we will screen the patient and find a wound, write our notes and say that we want to manage this patient with wound care. Then the following day when you go, the surgeons have taken over the patient and maybe the patient is already prepared for theatre. This makes me feel they think we don't know our work.” —Provider 21, podiatrist

#### Poor Communication

Poor communication affected both vertical and horizontal collaboration. This occurred at 2 levels: between providers at the tertiary hospital and those at PHC, and among health care providers in different specialty clinics at the tertiary hospital. For instance, nurses reported that some doctors at a tertiary hospital down-referred patients to PHC clinics in Soweto, without a detailed report on further management at the PHC level. This led to most patients being referred back to a tertiary hospital, especially when nurses at PHC did not know how to manage the patients. In addition, providers at the tertiary hospital rarely communicated with each other when managing patients with multimorbidity; some providers would provide a double prescription to patients with comorbid conditions especially those that cluster together (such as T2DM and hypertension) without realizing that the patients had already received similar prescriptions in a different specialty clinic. This led to cases of oversupply of medication, drug duplication, and mismanagement of patients.

**Ethnographic case observation of communication problems.** Patients with T2DM and other comorbidities came to the clinic with unused medications. There were empty drawers at the clinic reception where all unused medication were kept. Sometimes, patients would return the unused medication to the pharmacy when they came to collect new supplies. Nurses would shout at the patients for being nonadherent to their treatment. After a series of observations and informal conversations with patients, it became clear that patients were given extra medications especially when they attended other clinics for comorbidities.

One nurse expressed her amazement when she said:

*When they come to the clinic, you will be surprised that they still have enough medication for another month or so.* —Provider 4, nurse

Probing further, a doctor revealed circumstances in which oversupply of medication occurred:

*The patient has a file in dermatology, he gets his medication and then when he come for his diabetic clinic, it's a different file that they have and there is no communication between the two clinics. In such cases, double prescriptions may occur*. —Provider 10, doctor

Poor communication also led to treating diseases in isolation from the other conditions among individuals with comorbidities:

*We try to ask if they have any other diseases but with workload, and if the other diseases are not indicated in the file, I just treat the disease I know of.* —Provider 3, doctor

#### Noncentralized Patient Information System

**Ethnographic case observations of unlinked medical records.** The MOPD clinic had computers, but they were typically not linked to other departments due to network connectivity challenges. In most cases, data were captured manually. The diabetes/endocrine clinic is one of the 3 specialty clinics at the tertiary hospital that has implemented an electronic health record system (the Intellovate system). This system captures patient's information through biometrics. The system also scans through the patient's file every time they have a clinical encounter with the doctor. The up-to-date information captured in this clinic cannot be shared with other clinics because this system has not been rolled out to other clinics.

This case observation reveals that the lack of a centralized patient records system in a tertiary hospital challenged collaborative care efforts. This led to manual capture of patient's information through their files. Thus, providers complained of spending more time checking patient's files and the poor quality of care provided for patients requiring more than one clinic. In addition, some patients' files were lost at the hospital registry (Table 3).

#### Staff Shortage

Staff shortage and workload were reported to negatively affect care collaboration across disciplines:

*Collaboration is not that easy. If you find there is something urgent you have to address, and you have other patients in the queue waiting, this makes it difficult* …. —Provider 3, doctor

Staff shortage and workload were reported to negatively affect care collaboration across disciplines.

Another provider shared the same sentiments when she said:

*The problem is we have few doctors and many patients.* —Provider 1, nurse

In addition, integrating services and working as a team was also a challenge due to the existing professional hierarchical structures for managing patients:

*A podiatrist is not exactly authorized to prescribe antibiotics. When the doctor is not around maybe once they've left the clinic when they finish, you find that it becomes a challenge. Because I always need a medical doctor to prescribe certain drugs for me.* —Provider 14, Podiatrist

Interprofessional conflicts were also highlighted as another reason why collaborative care was not working well (Table 3).

Proximity to other clinics was also reported to negatively influence collaborative care. For example, HIV, renal, and psychiatric clinics were said to be meters away from the diabetes/endocrine clinic:

*Some clinics like psychiatric are far away […] It is difficult to start looking for a psychologist when they cannot be reached by telephone.* —Provider 7, doctor

### Patient Support and Involvement in Care

Patient support was said to be imperative especially for those who managed more than one chronic condition. However, it was mentioned that due to workload, providers never had enough time to provide personalized care to patients:

*We try to involve them [patients], but the workload is too much, we have not much time for this*. —Provider 16, doctor

Support was mostly done in group forums, such as during the diabetes education sessions. Yet, this support was not always accessed, especially when some patients had attended such sessions before or when they were in a hurry to join queues in other clinics.

*They are always in a hurry to leave the education class. They will complain that they are getting late for other clinics.*—Provider 6, nurse

*They [patients] feel it is a waste of time because they have been in the sessions before.* —Provider 17, diabetes nurse educator

Another key challenge for patient support was a language barrier. Elsewhere, we have extensively described how language barriers hinder health care providers from managing patients and providing patient-centered care.^36^

**Ethnographic observation during an ongoing diabetes class.** In a diabetes class, the dietician was busy educating patients about the permissible and nonpermissible foods, monitoring blood glucose, and self-care. Seven patients were seated around a large table that was positioned at the center of the room. Three patients were very active, asking questions and discussing their experiences managing diabetes. Three others were partly engaged, while 2 patients were very quiet and seemed disinterested. Suddenly the dietician pointed at 1 quiet patient and asked, “Mama [mother], why are you not saying anything?” The woman did not answer, but only stared at the dietician. One other woman seated next to the quiet woman quickly said, “She is from Mozambique and does not understand either English or the local languages.” The dietician, sounding empathetic turned to me and said, “This is so sad. Language barrier is a key challenge that we experience especially with patients from Mozambique.” The dietician paused for a few minutes, then proceeded with educating the other patients. The patient from Mozambique stared at the pictures and charts hanging on the wall.

Moreover, although patients' family or caregivers were invited to join diabetes education sessions, this was challenging for patients who live alone or who don't have family.

Similarly, social workers reported some of the challenges they experienced while involving family members in patient's care:

*Sometimes, we don't receive a good welcome from the patient's relatives or other caregivers.* —Provider 19, social worker

*We make all the family and patient sit down and talk about the patient's condition […] the problem comes when they [family] abandon the patient and don't want to be involved in the care.* —Provider 20, social worker

## DISCUSSION

This ethnographic study investigated integrated and collaborative chronic care at a tertiary hospital in Soweto to examine how the health care system functions to manage patients with comorbid T2DM and HIV. We found that patients with comorbid T2DM and HIV visited different clinics with limited collaboration among health care providers within the different clinics at the tertiary hospital and with PHC clinics in Soweto. This situation did not arise because of a lack of will from health care providers; rather, it reflected health system deficiencies such as staff shortage, noncentralized record keeping, poor communication, and medication stock-outs. Lack of communication impeded people from addressing major health concerns. In addition, patients with more advanced T2DM symptoms who arrived at the MOPD at the tertiary hospital were required to seek care at this level first. Then, they were referred to the diabetes/endocrine clinic only when their condition worsened. This process often delayed more specialized care for about 2 months and arguably made them sicker. In what follows, we address some of the major findings around care provision for patients, and we make recommendations for improving the care of patients with multimorbidities in Soweto and in similar settings in low-and middle-income countries (LMICs).

Patients with co-occurring chronic illnesses require complex models of care, involving integration and collaboration of services among professions and institutions. South Africa has already implemented the ICDM model to strengthen PHC facilities to care for patients with chronic multimorbidities.^12^ However, this model has not been implemented in most PHC clinics in Soweto. Although patients with HIV can be managed at PHC clinics, those with T2DM are mostly referred to a tertiary hospital.^37^ This is because of systemic challenges such as staff shortages, untrained nurses at PHC clinics, lack of equipment and medical supplies, and poor outcomes. Such systemic barriers were also found in a Cape Town study that reported 2 separate clinics for patients with T2DM and HIV even within PHC clinics.^15^ In addition, our findings may provide useful insights into why a task sharing program that aimed to increase nurses' capacity to manage and offer prescriptions for NCDs did not result in intensification of treatment for these diseases.^11^ Thus, it is clear that reorganization of PHC according to the ICDM model is still experiencing challenges throughout the country.

Patients with co-occurring chronic illnesses require complex models of care, involving integration and collaboration of services among professions and institutions.

Furthermore, the spaces in which chronic care is differentially provided matters. Atun et al.^29^ argued that these clinical arrangements are essential to the health system and the context where interventions are being implemented. It is therefore important to recognize that integration of chronic care models is impeded at the level of the health system in public health facilities in South Africa. Studies conducted in other low-resource settings have found that the readiness of health services scale-up for the management of chronic conditions through an integrated chronic care approach failed due to lack of staff, lack of access to treatment protocols, inconsistent supply of essential drugs, and other systemic barriers.^38^^–^^40^ Such findings align with what was found at the tertiary hospital in Soweto.^36^^,^^37^ To bolster integration of chronic care through the health system therefore requires more investment in detection, diagnosis, treatment of NCDs alongside and in tandem with HIV care at the PHC level.^11^ This will reduce cost, improve care, and enhance patient outcomes.

Care pathways through a well-structured referral system are required for integrated care for patients with multimorbidities.^17^ The current study revealed several limitations in both up- and down-referral. For instance, some patients walked into the tertiary hospital without a referral letter, while some doctors and nurses referred patients who did not qualify for a referral to the tertiary hospital. All these gaps and frequent bypassing of a structured referral system led to congestion and long queues at the tertiary hospital, contributing to overextending its services. Indeed, most health care providers revealed that some of the patients seen at the tertiary hospital could have easily been managed at the PHC clinics, further emphasizing the point that more investment in human resources is needed for PHC. Mojaki et al.^41^ have reported similar findings, whereby most patients seen at the MOPD and casualty had bypassed the referral system. More than half of the patients seen at these units could have been managed at the PHC facilities, a finding similar to a report from King Edward VIII Hospital in Durban^42^ and a factor that is overburdening tertiary care and leading to high cost.^43^ In addition, our findings are in line with an ethnographic study conducted in Guatemala that found that health system challenges, including hospital bureaucracies, communication breakdowns, and fragmented care, were key restricting factors that hindered patients from accessing care.^44^ Atun et al.^29^ similarly describe concern for how these communication networks break down to influence the rate at which an intervention is integrated into the general health system. Thus, targeted focus on identifying and addressing referral cogs in the health system may lead to improved and integrated NCD-HIV care, as well as improved health outcomes of vulnerable populations.

Care pathways for patients through a well-structured referral system are required for integrated care for those with comorbidities.

Integrated care at the PHC level may alleviate many of the challenges patients with multiple chronic conditions face at the tertiary hospital. Because most clinics were specialized, collaboration of services or task sharing^19^ was limited and care was focused on only one disease at a time. These findings are similar to other studies in low-resource settings that revealed how service provision for T2DM remains very limited at PHC, with services being offered in isolation in hospitals and at higher levels of care.^33^^,^^39^^,^^40^ Yet, important management issues impeded a more integrated chronic care approach for many patients: poor communication between clinicians and patients, poor communication among health care providers, and interprofessional conflicts and competition among specialized clinics. Some providers were not aware that patients had comorbidities alongside the immediate disease for which they were being treated. As such, the providers treated one condition in isolation from any others. Importantly, although diseases that cluster together such as T2DM and hypertension were managed together, patients had to visit separate clinics for any other additional conditions that they had. This finding concurs with recent studies among patients with multimorbidities in South Africa that have revealed that patients with concordant conditions (similar in risk profile and management) were more likely to progress further along the care continuum, while those with discordant multimorbidities (not directly related in pathogenesis or management) tended not to progress beyond diagnosis.^45^^,^^46^ We also found that even in cases in which clinicians were aware of other comorbidities, some ended up giving a double prescription to patients. Lack of a centralized patient information led to parallel care, drug duplications, and disjointed care between providers and patients, similar to other reports.^47^^–^^49^

Ultimately, health care providers rarely provided personalized support to patients or their caregivers due to workload, language barriers, and time constraints, similar to what we have reported elsewhere.^36^^,^^37^ This situation occurred despite many health care providers recognizing the need for personalized support; health systems inherently contained barriers that impeded this integrated care. Patient support was provided apart from clinical interactions and primarily in group forums. The most common type of forum was diabetes education sessions, with many patients attending only one session. Patient activation in groups was limited given the lack of personalized engagement with providers that would facilitate interactions and partnership in care.^36^ These findings may explain why a randomized controlled trial that evaluated the effectiveness of group education sessions in Cape Town, found no significant improvement in any of the primary or secondary outcomes after 12 months, apart from a significant reduction in mean systolic and diastolic blood pressure.^50^ Social workers also experienced challenges with families who were uncomfortable, disengaged, or absent. A growing body of research highlights the importance of meaningful engagement with families in clinical practice and a refocus on the providers' contribution in supporting families.^51^ Other studies in South Africa have also attributed low motivation to attend diabetes education sessions to poor patient–provider interaction, fear, dishonesty, and provider burn-out.^52^ Chronic conditions such as T2DM, however, require significant participation by informed patients, which may necessitate an ongoing collaborative process between patients and professionals to optimize long-term outcome. Yet, it is imperative to note that while the rationale for group education remains strong, such sessions must take into consideration the contexts in which they are implemented. Thus, future interventions on education forums for patients should be adapted for the infrastructural limitations and logistical barriers to patient retention.^50^

The ICDM model is proactive and well-coordinated multidisciplinary care, designed to improve collaboration between patients, providers, and caregivers.^12^ To achieve this goal in Soweto, PHC clinics must be strengthened in terms of providing adequate equipment and medication and training nurses to manage patients with T2DM and other chronic comorbidities. Only should the most severe cases be referred up to the tertiary level. Enhancing PHC interventions within the health system, especially within this context, can save time for the patient, enhance engagement in clinical visits for the patient and their family members, and ensure that further symptoms or health conditions are diagnosed early and cared for holistically.^51^ Even as chronic care escalates,^53^ the ICDM model must incorporate the complexities associated with multimorbidity, especially HIV and NCD co-occurrence, to meet the emerging needs of the burgeoning number of patients with the concurrent diagnoses.

The ICDM model is patient-centered, proactive, and well-coordinated multidisciplinary care, using new technologies to improve collaboration between patients, providers, and caregivers.

### Policy and Research Implications

As South Africa's health care sector undergoes important reforms,^9^ numerous health systemic challenges remain, hindering the implementation of integrated and collaborative care models. In particular, integrating diabetes care into HIV care programs has yet to be fully achieved due to health systemic challenges. Walt and Gilson^54^ have argued that most health policies wrongly focus attention on the content of reform and neglect the actors involved in policy reform; the processes are contingent on developing and implementing change and on the context within which policy is developed. Atun et al.^29^ have similarly emphasized that new interventions should be viewed with caution or circumspection by multiple potential adopters, affecting the extent, pattern, and rate of their adoption. Thus, when thinking about how to integrate care for patients with HIV and NCDs in South Africa and other LMICs, we suggest that PHC clinics and providers must be considered as key actors of implementation for ICDM models. Without addressing major issues of detection, diagnosis, and integrated care at the PHC level, higher levels of care such as secondary or tertiary hospitals will continue to face enormous patient burden in the everyday management of multiple conditions that can and should be relegated to the PHC. Greater investment is needed in PHC clinics in terms of equipment, medication, and staff who can deliver the consistent, careful, integrated, and patient-centered care patients deserve.

The MOPD is the centralized clinic through which patients first initiate care at a tertiary hospital in Soweto. We recommend strengthening the MOPD to offer integrated and collaborative care to patients with T2DM and other chronic comorbidities, especially HIV. Moreover, a key feature of task sharing or collaborative care is a team-based approach to care,^19^ whereby specialists at tertiary hospital engage and communicate among themselves and with providers at PHC clinics to improve patient outcomes.^55^ In the context of this study, clinicians at the tertiary hospital rarely communicated with providers in different clinics within the hospital or at PHC clinics, largely due to staff shortages, limited time for meetings, and poor communication. Strengthening the MOPD and enhancing a proper collaborative care require training and increasing the number of staff, as well as implementing a centralized electronic data capture system at the tertiary hospital that will ease communication and task sharing among providers and patients across different levels of care. Further, addressing key systemic issues especially at PHC clinics will enable early screening, detection and management of comorbidities, ease workload at tertiary levels of care, and create a patient-centered as opposed to disease-specific approach.

Findings reported from our study have implications for other LMICs that are poorly prepared to manage multimorbidity, partly due to inadequacies in the health system infrastructure (shortages of trained health care providers, equipment, and medication).^56^^,^^57^ For effective implementation and sustainability of the integrated care models, countries must adapt such models to fit their context. This tailoring calls for research into how countries are operationalizing and implementing integrated care and what challenges and opportunities exist. Such research may facilitate creating context-specific interventions or strategies that would enhance successful implementation of integrated care models. It may include assessing issues of human resources for health, equipment and medication, sustained decision support, developing comorbidity guidelines and checklists, and so forth.^58^ Importantly, the term *multimorbidity* does not seem to have a universally accepted definition.^2^ It is imperative to develop a standardized definition that can be incorporated into research agendas to identify the evidence gaps and to inform the organization of health systems.

For proper and sustainable implementation of the integrated care models, countries must adapt such models to fit their context.

Moreover, health care providers working with chronic care patients in LMICs must align chronic care to meet the needs of the patients and the population at large. This alignment will require a properly functioning patient information system to facilitate care coordination and effective communication between providers and patients. Establishing community linkages in care is one of the key tenets of integrated and collaborative care. Thus, a need exists to develop stronger links with communities to promote awareness on chronic comorbidity or multimorbidity prevention strategies through approaches such as outreach programs, early screening, self-management, and self-care.

Lastly, the coronavirus 2019 (COVID-19) pandemic has illustrated the need to understand how multimorbidities create vulnerabilities and interact with new infections (for example HIV and TB and immunosuppressed population groups). Thus, a need exists for more research to understand the complexities around multimorbidities and health systems to be better prepared to provide more effective care.

### Limitations

This study did not involve PHC facilities in Soweto and thus excludes what happens in these settings. Yet, our ethnographic interviews encompassed observations and informal conversations with different actors in the health care system that inform what challenges and opportunities are present within these settings. This information is especially relevant regarding patients who had been referred from PHC to tertiary hospital and provided insights about their experiences with PHC. Further, findings from this study concur with other qualitative studies^10^^,^^14^^,^^15^ that have focused on care provisions for patients with T2DM, HIV/AIDS, and other comorbidities in PHC in South Africa. This being an ethnographic study, issues around reflexivity and subjectivity were considered. First, being an outsider (non-South African) may have influenced the primary researcher's views on care provision. Second, the researcher relied on her own interpretation which could be biased. However, a constant thoughtful process in reviewing field notes, observations, and interviews with the participants and other researchers involved in this study allowed flexibility in data collection, analysis, and reporting of study findings.^59^ In this way, reporting of research finding was objective, which increased scientific rigor.

## CONCLUSIONS

Challenges experienced by patients with T2DM, HIV, and other chronic comorbidities in Soweto call for new ways to improve patient care by thinking and acting among policy makers, health care organizations, and health care professionals—as well as patients and caregivers. In addition to investing in and strengthening PHC-level disease detection, diagnosis, and care, we recommend strengthening the MOPD at the tertiary hospital to offer integrated and collaborative care to patients with T2DM and other comorbidities. The MOPD should also work with PHC clinics to ensure that patients can receive reliable care closer to their homes and families. To achieve this, health policy makers must address health system challenges such as lack of medical supplies, staff shortages, and a centralized patient information system in the public health care system. Improving the information that health care providers have from the level of the PHC clinic to the MOPD and specialty clinics is imperative, not only to improve the implementation of policies aimed at strengthening the health care system in South Africa but also to ensure the sustainability of such policies.

## Notes

**Peer Reviewed**

**First published online:** February 12, 2021.
